# Rescuing axons from degeneration does not affect retinal ganglion cell death

**DOI:** 10.1590/1414-431X20155106

**Published:** 2016-03-18

**Authors:** S. de Lima, B.S. Mietto, C. Paula, T. Muniz, A.M.B. Martinez, P.F. Gardino

**Affiliations:** 1 Laboratório de Neurobiologia da Retina, Centro de Ciências da Saúde, Instituto de Biofísica Carlos Chagas Filho, Rio de Janeiro, RJ, Brasil; 2 Laboratório de Neurodegeneração e Reparo, Departamento de Patologia, Faculdade de Medicina, Centro de Ciências da Saúde, Universidade Federal do Rio de Janeiro, Rio de Janeiro, RJ, Brasil

**Keywords:** Optic nerve crush, Calpain inhibitor, Axon preservation, Axon degeneration, Retinal ganglion cells, Cell survival

## Abstract

After a traumatic injury to the central nervous system, the distal stumps of axons undergo Wallerian degeneration (WD), an event that comprises cytoskeleton and myelin breakdown, astrocytic gliosis, and overexpression of proteins that inhibit axonal regrowth. By contrast, injured neuronal cell bodies show features characteristic of attempts to initiate the regenerative process of elongating their axons. The main molecular event that leads to WD is an increase in the intracellular calcium concentration, which activates calpains, calcium-dependent proteases that degrade cytoskeleton proteins. The aim of our study was to investigate whether preventing axonal degeneration would impact the survival of retinal ganglion cells (RGCs) after crushing the optic nerve. We observed that male Wistar rats (weighing 200-400 g; n=18) treated with an exogenous calpain inhibitor (20 mM) administered via direct application of the inhibitor embedded within the copolymer resin Evlax immediately following optic nerve crush showed a delay in the onset of WD. This delayed onset was characterized by a decrease in the number of degenerated fibers (P<0.05) and an increase in the number of preserved fibers (P<0.05) 4 days after injury. Additionally, most preserved fibers showed a normal G-ratio. These results indicated that calpain inhibition prevented the degeneration of optic nerve fibers, rescuing axons from the process of axonal degeneration. However, analysis of retinal ganglion cell survival demonstrated no difference between the calpain inhibitor- and vehicle-treated groups, suggesting that although the calpain inhibitor prevented axonal degeneration, it had no effect on RGC survival after optic nerve damage.

## Introduction

After an insult, neurons from the adult optic nerve do not spontaneously regenerate their axons beyond the injury site (1). Recently, our group demonstrated that long-distance axonal regeneration was achieved when retinal ganglion cells (RGCs) were exposed to macrophage-derived factors along with genetic deletion of the phosphatase and tensin homolog gene and increased levels of cyclic adenosine monophosphate in those neurons (2). Thus, the adult optic nerve serves as an invaluable model system with which to study the key mechanisms that suppress or promote neural repair after central nervous system damage (3). Optic nerve crush is followed by the degeneration of affected axons and RGC death in a progressive and stereotypic process termed Wallerian degeneration (WD) (4 5 6). Axonal fragmentation is an early event during WD. In contrast, RGC death occurs only at later stages, approximately 14 days after injury, when about 15-20% of remaining RGC neurons are observed in the retina (2,5,7,8). Notably, at day 7 after injury, while RGCs are still viable and show characteristics indicative of the regenerative process, all crushed axons at the distal stump have already undergone full degeneration (4,7,9). This axonal disintegration process is mediated by the activation of calcium-dependent proteases, such as calpains, that target cytoskeleton proteins, especially neurofilaments, for degradation (10).

Calpains are ubiquitously expressed in the central and peripheral nervous systems and are broadly activated when the intracellular calcium concentration rises to a standard level threshold (11). Calpain activation is observed not only after a traumatic injury but also during the course of neurodegenerative diseases (12 13 14 15). Previous studies from our group and others have shown that inhibition of calpain activation results in a certain degree of axonal protection after injury (16,17). However, whether calpain inhibition affects RGC survival after optic nerve crush injury has been rarely investigated. Here, we provided morphological evidence that exogenous administration of a specific calpain inhibitor delays WD-induced axonal degeneration of optic nerve fibers for up to 4 days. However, inhibiting axonal degeneration was not sufficient to rescue RGCs from death.

## Material and Methods

### Optic nerve surgery

All procedures and protocols for the use and care of animals were approved by the Ethics Committee for the Use of Experimental Animals at the Universidade Federal do Rio de Janeiro. A total of 18 male Wistar rats weighing 200-400 g were used in this study. The rats were deeply anesthetized with intramuscular injections of ketamine:xylazine (100:15 mg/kg), and the left optic nerve was carefully exposed under a dissection microscope. Optic nerve crush was performed using a vascular clip (Kent Scientific, USA) with 10 g of force for 30 s, 1 mm distal from the eye. Care was taken to avoid causing damage to the ophthalmic vessel (18). Soon after the crush injury, the dura matter was opened and Elvax complexed with either a calpain inhibitor or its vehicle was inserted. The right optic nerve was used as a control.

### Elvax preparation

An ethylene-vinyl acetate copolymer resin (Elvax 40W, DuPont, USA) was used to administer the calpain inhibitor or vehicle. The resin preparation consisted of 35 µL of Fast Green, 25 µL of a calpain inhibitor (calpain inhibitor-2, Mu-F-HPh-FMK; Kamiya Biomedical Company, USA) at a concentration of 20 mM, and 20 µL of dimethyl sulfoxide. The calpain inhibitor, a peptide-fluoromethyl ketone, was chosen for this study because it causes irreversible inhibition and is nontoxic to cells. Before implantation, the resin was sectioned using a cryostat to a thickness of 60 µm and a length of 250 µm. Control resin was made following the same protocol, but the control resin contained only the vehicle, dimethyl sulfoxide.

### Preparation of tissue for transmission electron microscopy

Four days after surgery, 8 animals were deeply anesthetized and perfused through the heart with a solution containing 2% paraformaldehyde and 4% glutaraldehyde in 0.1 M phosphate buffer (pH 7.4). A 2-mm segment distal to the crushed site was dissected and processed for light and electron microscopy. Segments from the same region of the non-operated eye were obtained as a control. All segments were postfixed for 1 h in 1% osmium tetroxide plus 0.8% potassium ferrocyanide and 5 mM CaCl_2_ in 0.1 M cacodylate buffer. The segments were dehydrated in increasing concentrations of acetone (from 15 to 100%) and finally embedded in Embed 812 resin (Electron Microscopy Sciences, USA). Semithin and ultrathin sections were obtained using an RMC MT-6000 ultramicrotome. Semithin sections (1 μm) were stained in 1% toluidine blue and observed under a light microscope. Ultrathin sections (60 nm) were collected on copper grids and stained for 30 min in uranyl acetate followed by 10 min in lead citrate. Electron micrographs were acquired on a Zeiss 900 transmission electron microscope (Germany).

### Quantitative analysis

Ten images from the ultrathin cross sections from each optic nerve were systematically captured using a transmission electron microscope at a magnification of ×7000. This systematic method globally scanned and automatically sampled the sectioned area of the nerves, avoiding selection bias. In total, 40 images were obtained for each group. Morphometric analysis was conducted using the National Institutes of Health's software ImageJ (USA). The following parameters were measured: number of preserved fibers, number of degenerating fibers, and G-ratio (the ratio of the axonal diameter to the fiber diameter). All morphometric parameters were statistically analyzed with use of Prism software (Graph Pad Inc., USA). Data are reported as means±SD. A paired two-tailed *t*-test for parametric data or a two-tailed Mann-Whitney *U*-test for non-parametric data was performed. Data were considered significant at P<0.05.

### Immunohistochemistry of flat-mounted retinas

For flat-mounted retina preparations, 10 animals were euthanized using an anesthetic overdose; then the retinas were gently dissected and incubated in 4% paraformaldehyde for fixation for 1 h. The retinas were then transferred to culture vials, subjected to two washes of 15 min each in phosphate-buffered saline (PBS), and incubated in blocking solution containing 5% bovine serum albumin plus 0.25% Triton X-100 in PBS for 1 h. The retinas were then incubated overnight with a primary antibody to βIII-tubulin (Tuj1, 1:500; Covance, USA). The following day, the retinas were washed twice in PBS and incubated in a secondary antibody (goat anti-mouse Alexa Fluor 488, 1:300; Molecular Probes) for 1.5 h at room temperature. Later, retinas were flat mounted onto a glass slide, coverslipped, and mounted with N-propyl gallate for analysis using a fluorescence Zeiss microscope.

### Quantitative analysis of RGCs

The total number of Tuj1-immunopositive RGCs were manually counted in four fields of 0.325 mm^2^ each, at a magnification of ×20, using the software ImageJ (USA). The results were statistically analyzed using one-way analysis of variance with Tukey's *post hoc* tests. Values of P<0.05 were considered to be significant.

## Results

**Figure 1:**
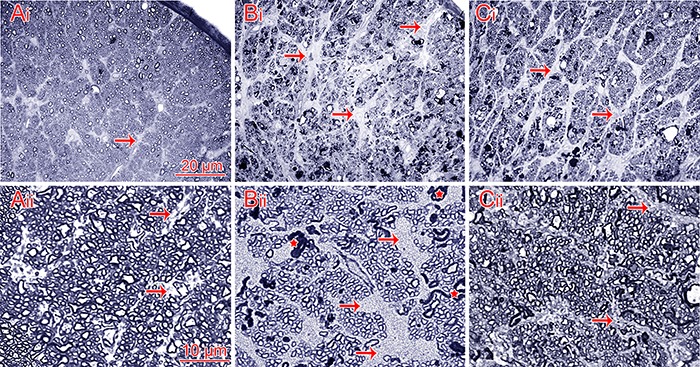
Semithin cross sections of rat optic nerves. *Ai* and*Aii*, Normal optic nerve having several bundles of nerve fibers among septa within astrocytic processes (arrows). *Bi*and *Bii*, Crushed optic nerve without calpain inhibitor treatment. There is intense astrogliosis (arrows), the nerve fiber bundles are more dispersed, and degenerating fibers are clearly observed (stars).*Ci* and *Cii*, Crushed optic nerve with calpain inhibitor treatment. There is less gliosis (arrows) and the cytoarchitecture of the nerve is better preserved. Scale bars: *Ai, Bi*, and *Ci,* 20 µm; *Aii*,*Bii*, and *Cii,* 10 µm.

Considering that optic nerve crush triggers axonal degeneration through calpain activation during the first 24 to 72 h after injury (19,20), we first assessed, by light microscopy, whether inhibiting calpain activation would prevent axons from entering the active process of self-destruction. Normal fibers from uninjured optic nerves are shown in Figure 1Ai and Aii. At 96 h after the nerve crush, the vehicle-treated group showed a marked disorganization of the optic nerve structure, along with several fibers undergoing degeneration and an intense astrogliosis reaction (Figure 1Bi and Bii). By contrast, inhibiting calpain led to better organization of the optic nerve cytoarchitecture, which was followed by noticeable preservation of nerve fibers and less astrogliosis (Figure 1Ci and Cii). Therefore, our preliminary data indicated that a calpain inhibitor administered during the early stages of WD prevented axonal degeneration compared with vehicle-treated nerves subjected to the same trauma.

**Figure 2:**
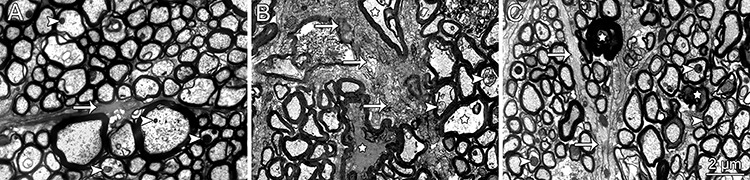
Ultrathin cross sections of optic nerves. *A*, Normal, uninjured nerve with fibers showing preserved axoplasm, mitochondrial profiles (arrowheads), and astrocytic septa (arrow). *B*, Lesioned, vehicle-treated optic nerve showing dark and watery (stars) degenerating nerve fibers, fibers with anomalous myelin alterations, thick astrocytic septa (arrows) and swollen mitochondrial profiles (arrowheads).*C*, Lesioned, calpain-inhibitor treated optic nerve showing less astrogliosis (arrows) and better overall organization of the nerve, with some nerve fibers that appear normal (arrowheads) and some that are degenerating (stars). Scale bar: 2 µm.

To substantiate this finding, we examined ultrathin sections from injured and uninjured optic nerves and compared their morphological features with those of the damaged nerves derived from the treated group. The ultrastructural appearances of the optic nerves are shown in Figure 2. The normal, uninjured nerve fibers obtained from rats showed preserved axoplasm, healthy mitochondrial profiles, and astrocytic processes with regular thicknesses (Figure 2A). However, after optic nerve crush injury, dark- and watery-type axonal degeneration was observed together with other fibers having an anomalous myelin sheath in the vehicle-treated group (Figure 2B). We also observed thick astrocytic septa and swollen mitochondrial profiles, all indicative of an advanced stage of WD. By contrast, nerves that received the calpain inhibitor presented less astrogliosis and showed better organization of nerve fibers bundles. Dark-type degenerating axons and fibers with anomalous myelin were observed, albeit to a lesser extent than that observed in injured, vehicle-treated nerves (Figure 2C).

**Figure 3:**
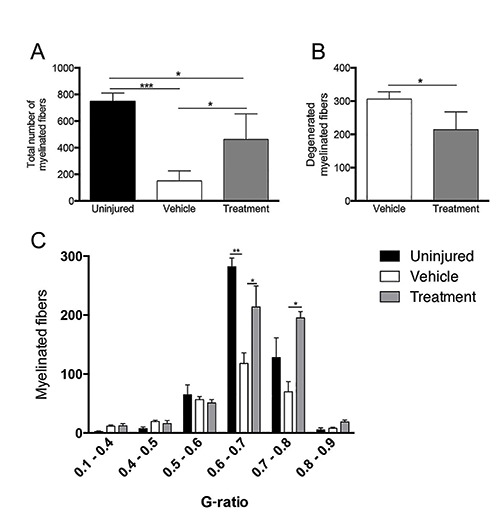
*A*, Quantification of optic nerve myelinated fibers. Data are reported as means±SD. *B*, Quantification of degenerated optic nerve fibers in calpain- and vehicle-treated injured animals. Data are reported as means±SD. *C*, Analysis of the G-ratio, showing distribution of fibers in different ranges. One-way ANOVA with Tukey's*post hoc* tests was used for statistical analysis (*P<0.05, **P<0.01, ***P<0.001; n=4 per group).

Morphometric studies were conducted on calpain inhibitor- and vehicle-treated injured optic nerves 4 days after injury. Our quantitative analysis showed a significant increase in the number of preserved myelinated fibers in the calpain inhibitor-treated group compared with those in the vehicle-treated group (Figure 3A). As expected, significantly more degenerated nerve fibers were observed in the vehicle-treated than inhibitor-treated nerves, suggesting an advanced stage of nerve degeneration in the group of animals that did not receive the calpain inhibitor treatment (Figure 3B). With regard to the G-ratio, the inhibitor-treated nerves also showed better results, because most fibers had G-ratios within the range of normal optic nerves (0.6-0.7 and 0.7-0.8; Figure 3C).

**Figure 4:**
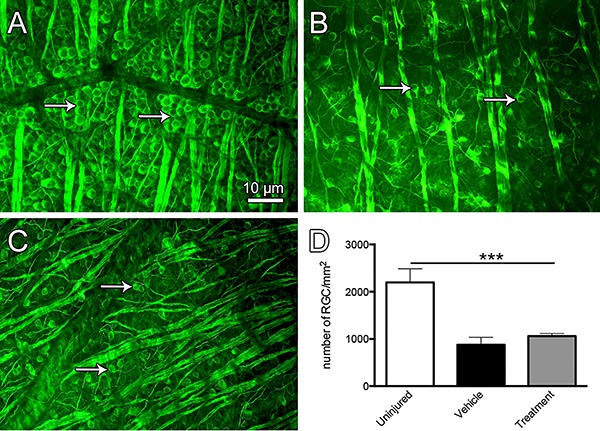
Results of βIII-tubulin (Tuj1) immunofluorescence labeling of retinal ganglion cells (RGC) in flat-mounted retina preparations. The number of neuronal cell bodies (arrows) in the normal nerve (*A*) was greater than that in animals that were lesioned and treated with vehicle (*B*) or a calpain inhibitor (*C*). Quantification of Tuj1 as determined by the number of immunofluorescent RGC/mm^2^ (*D*). *P<0.001, one-way ANOVA and Tukey's post-test. Scale bar: 10 µm; n=5 per group.

We next aimed to verify whether blocking axonal degeneration by preventing calpain activation affected RGC survival. Thus, we examined βIII-tubulin immunofluorescence to visualize RGCs in flat-mounted retinas obtained 4 days after nerve crush injury.Figure 4A is representative of a normal, uninjured rat retina and shows several RGCs. As expected, after injury, we observed a gradual decline in the number of viable RGCs in the vehicle-treated group 14 days after the trauma (Figure 4B). The retinas obtained from the calpain inhibitor-treated group also showed a marked reduction in the number of RGCs (Figure 4C). Our quantitative analysis showed that the number of RGCs in the flat-mounted retina preparations 14 days post-crush injury were the same in vehicle- and inhibitor-treated groups (Figure 4D). This result indicated that, although axons were rescued from degeneration after calpain inhibitor administration, their respective cell bodies died 14 days post-injury.

## Discussion

Axonal disintegration is an early event that occurs during the process of WD. Massive cell death occurs within 2 weeks after injury, when 60 to 70% of RGCs die, and 1 month after injury, when the rate of cell survival falls to only 5% (21). Many studies have shown that, although it is possible to block cell death, this is not accompanied by axonal preservation or regeneration; for example, overexpression of Bcl-2 or caspase-3 inhibition after injury increases cell survival but has no effect on nerve regeneration (22,23). Trophic factors, such as brain derived neurotrophic factor and neurotrophin 4/5, are known to improve RGC survival but only partially protect these cells from death after nerve injury (24 25 26 27). Blocking axonal degeneration in genetically mutated animals does not have a positive effect on cell survival. Mice with delayed WD (28) carry a mutation that delays axonal degeneration after acute traumatic lesion. This shows that the deprivation of nutritive support from the cell body is not the sole cause of axonal destruction but is rather an intrinsic mechanism within the axons that also governs axonal destruction (29 30 31 32 33 34 35). However, the presence of this mutation does not guarantee that the neuronal cell body will be protected. After optic nerve crush or axotomy and in models of increased intraocular pressure, there is a delay in WD. Nevertheless, no delay or increase in the rate of cell survival was observed in these genetically mutated mice (9).

Despite many approaches that demonstrate effects either on cell survival or axonal regeneration alone, there are some treatments that can stimulate both simultaneously. The most effective approach to date is the combination of intraocular inflammation, increased levels of cyclic adenosine monophosphate, and deletion of the phosphatase and tensin homolog gene. This treatment promotes the activation of at least three distinct pathways that are responsible for cell survival and axonal regeneration and leads to full-length regeneration of the optic nerve after a crush injury, with partial functional recovery of simple visual reflexes (2,36 37 38).

We investigated the impact of an acute blockade of axonal degeneration on cell survival using an *in vivo* pharmaceutical approach by applying a calpain inhibitor at the site of the crush injury. The calpain inhibitor was embedded in a resin that released the inhibitor at a slow rate, generating an immediate to mid-term effect on axonal degeneration. We used a calpain inhibitor to prevent the degradation of cytoskeleton proteins, which is known to be a calpain-dependent event (17). We analyzed the tissue 4 days after injury because at that time fibers with intact axoplasm would exist with others that had already begun the WD process. Additionally, Agudo et al. (19) showed that the peak protein level for μ-calpain occurs 12-48 h after a central nervous system injury. We observed a treatment-induced delay in the rate of axonal degeneration 4 days after injury because at this time more fibers with intact axoplasm and fewer degenerated fibers were observed in the animals that had received the calpain inhibitor. Moreover, the G-ratios for those fibers were within normal parameters, suggesting that the fibers were capable of conducting proper action potentials.

In a recent report, Yang et al. (17) demonstrated that an endogenous calpain inhibitor, calpastatin, is a key mediator of axonal degeneration during development and after injury. Some groups have studied the effect of calpain inhibitors on RGCs and have demonstrated an increase in cell survival (14,39,40). The difference between our results and those from studies that observed an increased rate of cell survival may be explained by the methods used. We applied the calpain inhibitor to the injury site *in vivo*. In contrast, the other studies used *in vitro* approaches, applying the inhibitor directly onto RGCs; this approach may stimulate distinct intracellular pathways related to cell survival. Our approach relies on axonal transport to reach the cell bodies, whereas the *in vitro* approach acts directly on RGCs. In this study, we did not investigate the distal part of the axon; however, we believe that the same rate of degeneration would be observed as already shown by other investigators (9).

In conclusion, our results indicated that the delay of axonal degeneration 4 days after injury created a time window for the use of additional therapeutic strategies. For example, the prolonged use of calpain inhibitors combined with a pro-survival strategy may increase both axonal preservation and cell survival.
